# Looking on the Bright Side of Livestock Emotions—the Potential of Their Transmission to Promote Positive Welfare

**DOI:** 10.3389/fvets.2018.00218

**Published:** 2018-09-12

**Authors:** Luigi Baciadonna, Sandra Duepjan, Elodie F. Briefer, Monica Padilla de la Torre, Christian Nawroth

**Affiliations:** ^1^School of Biological and Chemical Sciences, Queen Mary University of London, London, United Kingdom; ^2^Institute of Behavioural Physiology, Leibniz Institute for Farm Animal Biology, Dummerstorf, Germany; ^3^Institute of Agricultural Sciences, ETH Zürich, Zürich, Switzerland; ^4^Department of Production Animal Clinical Sciences, Norwegian University of Life Sciences, Oslo, Norway; ^5^Centre for Proper Housing of Ruminants and Pigs, Agroscope Tänikon, Federal Food Safety and Veterinary Office, Ettenhausen, Switzerland

**Keywords:** affective states, emotions, emotional contagion, farm animals, wellbeing

## Abstract

Emotions can be defined as an individual's affective reaction to an external and/or internal event that, in turn, generates a simultaneous cascade of behavioral, physiological, and cognitive changes. Those changes that can be perceived by conspecifics have the potential to also affect other's emotional states, a process labeled as “emotional contagion.” Especially in the case of gregarious species, such as livestock, emotional contagion can have an impact on the whole group by, for instance, improving group coordination and strengthening social bonds. We noticed that the current trend of research on emotions in livestock, i.e., investigating affective states as a tool to assess and improve animal welfare, appears to be unbalanced. A majority of studies focuses on the individual rather than the social component of emotions. In this paper, we highlight current limitations in the latter line of research and suggest a stronger emphasis on the mechanisms of how emotions in livestock are transmitted and shared, which could serve as a promising tool to synergistically enhance the welfare of all individuals within a group.

## What are emotions?

Emotions have been defined as short-term affective states elicited by internal and/or external events and are associated with synchronized physiological, behavioral, and cognitive components (1, 2). The framework proposed by Mendl et al. (3) suggests that different aspects of the emotional experience (e.g., neurophysiological, behavioral, and cognitive components) of non-human animals can be assessed along two dimensions, namely valence (negative or positive) and arousal [from low to high (4–6)]. One of the main functions of emotions is to prepare an individual to quickly select an appropriate response (i.e., approach or avoidance depending on the positive or negative valence of the emotion) in order to cope efficiently with its environment (1, 2). Moreover, affective states, i.e., emotions and longer-term affective states (mood), can alter the way in which an individual

perceives its environment, e.g., making it more cautious (pessimistic-like) after a fear-inducing event or more optimistic-like after a positive event [so-called cognitive bias; (7)]. Considering the evolutionary importance of affective states, scientists' most widely held opinion is that these states occur across different taxa including invertebrates (1, 8–11). Although the lack of verbal communication in non-human animals precludes our access to the subjective component of emotions (i.e., feelings), there is an array of quantifiable parameters that allow us to assess their physiological, behavioral, and cognitive components. For instance, an emotion experienced by an individual can lead to changes in its body posture or expressions [e.g., facial or vocal signals/cues; (11–14)]. Since these expressions can be easily detected by other individuals, it is plausible that emotions do not only operate at the individual level, but also at the group level.

## The social component in emotions

The communication of emotions to conspecifics can play a key role in the regulation of social interactions (e.g., for group defense, play, agonistic behavior, maternal nursing, mating competition). Moreover, the expression of emotions can lead to the sharing of affective states between individuals (15). In gregarious species, synchronized emotional states within a group of individuals can be highly adaptive (16–18) and this phenomenon has been suggested to be a crucial element in the evolution of empathy (19, 20). The benefits of sharing emotional states between individuals include improved group coordination and strengthening bonds between individuals (21–24).

Different levels in the transmission of emotions have been proposed, with mechanisms requiring less cognitive load such as emotional contagion, and more cognitively sophisticated processes such as perspective taking and targeted helping (20, 25). Emotional contagion occurs when the affective state of an individual is influenced by the perception of the affective state of another individual (20). It results in state-matching between two individuals (e.g., distress with distress), without necessarily requiring conscious and effortful processing or self-other distinction (26). In the case of emotional contagion, the response of the subject should be in line with (i.e., match) the emotional state of the observed individual. By contrast, in the case of cognitive forms of empathy (“cognitive empathy”; e.g., perspective taking), the result will not necessarily be a matching state between the observer and observed individuals, because this phenomenon will result in the observer regulating its own emotional response in order to efficiently interact with the latter (27).

According to the Perception-Action Model of empathy proposed by Preston and de Waal (20), and as shown by neurobiological studies in humans (28), the attentive perception of the observed individual's emotional state automatically activates the observer's representation of this state. These representations trigger associated autonomic and somatic responses and allow the observer to connect with the internal state and situation of the observed individual through the activation of the neural representations of similar internal states that the observer has previously experienced (29–31).

## Evidence for transmission of emotions in livestock

Empathic responses occur widely within the mammalian taxon, with emotional contagion being the most common phenomenon investigated (24, 32). But since the field of non-human emotion research started to expand a few decades ago (33), research on farm animal welfare has focused mostly on the expression of emotions at the individual level, as an indicator of animals' welfare state [i.e., their physical but also psychological wellbeing; (24)]. By contrast, only a few studies have focused on emotional contagion and how the expression of emotions affects the welfare of the group (26, 34). The limited available evidence, however, suggests that this phenomenon might have a crucial impact on animal wellbeing (24). For instance, when pigs were restrained in a dispenser without access to food, other pigs later avoided the system/dispenser, especially when the reaction of being restrained was very aversive and associated with urination (35). In another study, untrained pigs showed a higher rate of defecation or higher levels of play when they observed conspecifics that were trained to anticipate an aversive or rewarding event, respectively (36). In addition, piglets showed a stronger reaction (higher proximity, decreased locomotion, and increase freezing behavior) toward distressed conspecifics when they had previously experienced the same stressor themselves (i.e., being restrained) compared with piglets that had not been restrained (34). In cattle, the presence of a stressed companion animal led to an increase in cortisol, a longer latency to feed and slower feeding rates in the tested subject, indicating increased fearfulness (37). Additionally, cattle showed a longer lasting approach response when a novel object was impregnated with urine of stressed conspecifics compared to urine of non-stressed conspecific. The increased fearfulness thus seemed to be at least partly mediated by olfactory cues present in the distressed animals' urine (37).

Vocalizations in particular have been shown to reflect emotional states in many species, and might therefore serve as a crucial channel for emotional contagion (15, 38). For example, pig vocalizations elicited during different stressful situations were related to the specific type of stress (14). Similarly, pig vocalizations, but also physiology, were affected by the induced emotional valence of repeated moderate aversive and rewarding events and are linked to emotional reactivity within and across different contexts (39). However, a similar experiment showed that when pigs heard recordings of distress calls from unfamiliar pigs of the same age and sex, the emotional valence of the calls did not induce a comparable state of distress (40). More recently, a study investigating the behavioral, physiological, and acoustic correlates of emotions in goats showed that parameters differed in each of these categories according to the valence and/or arousal of the emotions experienced by the animals (41). Subjects in high-arousal situations (such as food frustration), compared to low-arousal ones (such as isolation), showed lower heart-rate variability, higher respiration rates, increased body and head movements and vocalizations, and spent more time with their ears pointing forwards. In the positive situation (anticipation for food), compared to the negative ones (food frustration and isolation), goats spent less time with their ears oriented backwards and more time with their tails up. In addition, several acoustic parameters were identified as reliable indicators of arousal and valence. The fundamental frequency contour and energy quartiles of their vocalizations increased, while the first formant decreased, with arousal, whereas the fundamental frequency variation decreased from negative to positive valence (42). These results strongly support the notion that the vocal domain in group-living animals, such as goats, could be a potential way of emotion transmission. Moreover, playback experiments in goats indicated that subjects exposed to emotional-linked calls (food anticipation, food frustration, and isolation) from conspecifics preferentially showed a lateralized head-turning response to the right side (43). This right side head-turning bias suggests the involvement of the left hemisphere for processing calls conveying emotional contents (44, 45). In addition, it was demonstrated that the acoustic structure of domestic horses' whinnies varies between positive and negative contexts, and that familiar conspecifics are able to perceive this information (46, 47).

It is possible that lifetime experience could affect empathic responses. Very young subjects, or subjects housed in environmentally and/or socially deprived housing conditions, have shown impaired abilities to display emotional contagion (34, 48). In addition, the transmission of emotions can be enhanced by several factors, including familiarity and relatedness between the observer and the observed individuals, as well as former experience of a similar emotional situation by the observer (20). These context-specific ontogenetic differences warrant further investigations.

To date, the question of to what extent farm animals are able to perceive the emotions of conspecifics through behavioral, but also acoustic and olfactory cues and whether emotional contagion occurs as a result is still debated. However, considering the evidence for perception and contagion of emotions in several non-livestock taxa (15), farm animals are likely to also share these empathic capacities. More studies on emotional contagion in livestock are needed in order to extend our understanding of the impact of this phenomenon on animal welfare, and of the mechanisms underlying it.

## Disentangling modalities and limitations of the transmission of emotions

Emotional states in farm animals have been shown to be transmitted via olfactory cues (37), vocalizations (15), and direct observation of conspecific behavior (49). However, interpretations of current research on emotional contagion in livestock are often limited by two factors. First, the use of live animals as signalers does not control for other modalities than the ones primarily investigated (36). On the other hand, the restriction to only one signaling modality (e.g., acoustic cues through playbacks, visual cues through images or videos) does not provide a holistic view on the underlying mechanisms of emotional contagion. We thus encourage a multi-modal approach including controlled stimuli in the study of non-human emotional contagion, for example, by using signals produced by conspecifics experiencing fully validated positive and negative emotional states. A future step must be to investigate whether controlled visual (e.g., images or movies) or auditory cues (e.g., playbacks) alone and/or in combination can lead to the spread of affective states within the group. By providing signals of two or more modalities simultaneously, one could estimate whether cues simply add up or have synergistic effects, i.e., if and how this might enhance the transfer of emotions by making it more salient and/or relevant. In addition, violation of expectation experiments (i.e., providing cues that do not match an observer's expectations, such as displaying playbacks of positive valence and videos/images of negative valence simultaneously) could identify whether a subject forms a mental cross-modal representation not just about the features/appearance of another subject, but also about its emotional state (50, 51).

For a long time, the main aim of animal welfare research has been to reduce, and hence also to assess, negative emotions and to lower stress during an animal's life. Recent views, however, have pointed toward an effort to also explore and promote positive emotions (52, 53). Similarly, most studies investigating emotional contagion in livestock have focused solely on the transfer of negative emotional states. Yet, although the function of contagion of positive and negative emotions may differ, it is likely that the mechanisms underlying emotional contagion are independent of the valence (26). The bias toward research in negative emotions might be a result of the increased availability of parameters indicating negative compared to positive emotions (8, 9, 53). The increased set of tools developed to investigate negative emotions could be explained by the fact that many positive emotions are less intense in their expression compared with negative ones, and often the expression and perception of negative emotions (e.g., distress, need, pain) plays a substantial role for survival, making them more prominent and easily detectable (19, 54, 55). In contrast, the consequences of not being responsive to positive emotions expressed by conspecifics might be less severe regarding immediate survival.

Overall, we believe that there are several limitations in most of the existing studies on livestock emotions and emotion transfer, including a lack of validated and accurate assessments of the emotional state of both the producer (observed individual) and receiver (observer) of the emotional signal. Such validation could be done by using neuro-physiological, cognitive, or behavioral indicators of emotions (8, 42). In addition, there is a general lack of detailed evidence showing that the change in emotions observed in the receiver is due to the signal to which it was exposed to, and not due to other environmental cues that were not controlled for. To ensure that the changes observed in the animals are due to the signal, subjects should ideally be tested in a neutral environment (such as their home pen); an environment that does not induce an emotion by itself. Alternatively, the emotional state of the animal before exposing it to the signal should be assessed and controlled for.

## Implementation in applied settings and future directions

Emotional contagion can lead to the spread of both positive and negative emotions in groups of animals (35). This phenomenon is of strong importance for the welfare of group-housed domestic and/or captive animals. Indeed, emotional contagion could potentially be used as a tool to improve welfare by facilitating the spread of positive emotions as well as by reducing negative high-arousal emotions, or, at least, by preventing the spread of such emotions. Therefore, knowledge about the primary modalities that livestock species use to perceive emotional cues from conspecifics or even from humans, in case of cross-species contagion of emotions (56), would help us to better comply with their emotional needs and thus provide them with a better quality of life.

Vocalizations are a potent modality to express emotions as shown above. It would be interesting to test how conspecifics perceive emotion-linked calls and how these modulate the emotional state of the receiver. This knowledge could be used to design tools for improving welfare. For instance, playbacks of positive low-arousal vocalizations (or other sensory cues) could have the potential to decrease the impact of stressful events, such as transport, rehoming in an unfamiliar environment or veterinary practice, on the animal. We argue that positively valenced emotional stimuli (e.g., vocal or olfactory cues) could be used as a tool to promote positive emotions in receivers, or alternatively, to reduce negative ones. It would thus be valuable to investigate whether negative high-arousal states can be counteracted by using playbacks of positive low-aroused calls (or other sensory cues). Additionally, one of the hypothesized functions of positive emotions and their contagion is to strengthen social bonds (57). Tools such as social network analyses, which provide us with the quality (e.g., affiliative or agonistic) and quantity (number of interaction) of social relationships, could inform us on if and how negative and/or positive emotional stimuli spread within a group.

Given the widespread occurrence of emotional contagion in a diverse set of animal taxa, livestock species surely are no exception to the rule. The investigation of shared emotional states in livestock and its interactions with other social phenomena, such as social buffering (58), however, remains an underdeveloped field. In addition, several methodological limitations (e.g., the use of live animals as signalers or the restriction to one signaling modality in playback experiments) still have to be addressed, and there is a need for research to move away from negative emotions in order to include positive ones as well. Indeed, in order to harness the full potential of empathic responses in livestock and to transfer it into an applied setting, we must first identify the mechanisms and modes involved in the transmission of affective states. Understanding the perceptual mechanisms of the social dimension of animal emotions will open new ways to reduce high-arousal negative emotions and, in the long-term, promote positive welfare in livestock.

## Author contributions

All authors listed have made a substantial, direct and intellectual contribution to the work, and approved it for publication.

### Conflict of interest statement

The authors declare that the research was conducted in the absence of any commercial or financial relationships that could be construed as a potential conflict of interest.
